# Supplementary material for: Diagnostic performance of circulating tumor DNA as a minimally invasive biomarker for hepatocellular carcinoma: a systematic review and meta-analysis
Source: PeerJ. 2022 Nov 3;10:e14303. doi: 10.7717/peerj.14303 (PMC9637356; doi:10.7717/peerj.14303)
Supplement: Supplemental Information 4 [file peerj-10-14303-s004.docx]

| Support table 1. Characteristics of the included studies. | TN | 55 | 55 | 44 | 50 | 19 | 10 | 35 | 35 | 5 | 50 | 48 | 47 | 16 | 15 | 14 | 13 | 12 | 73 | 25 | 10 |
| --- | --- | --- | --- | --- | --- | --- | --- | --- | --- | --- | --- | --- | --- | --- | --- | --- | --- | --- | --- | --- | --- |
|  | FN | 21 | 10 | 15 | 31 | 24 | 23 | 15 | 38 | 16 | 38 | 28 | 15 | 21 | 21 | 10 | 19 | 19 | 73 | 2 | 21 |
|  | FP | 0 | 0 | 6 | 0 | 4 | 0 | 0 | 0 | 3 | 0 | 2 | 3 | 0 | 1 | 2 | 3 | 4 | 0 | 12 | 0 |
|  | TP | 4 | 15 | 10 | 14 | 22 | 17 | 49 | 26 | 16 | 12 | 22 | 35 | 5 | 5 | 16 | 7 | 7 | 6 | 23 | 14 |
|  | NLR | 0.84 | 0.40 | 0.68 | 0.69 | 0.63 | 0.58 | 0.23 | 0.59 | 0.80 | 0.76 | 0.58 | 0.32 | 0.81 | 0.86 | 0.44 | 0.90 | 0.97 | 0.92 | 0.12 | 0.60 |
|  | PLR | Infinity | Infinity | 3.33 | Infinity | 2.75 | Infinity | Infinity | Infinity | 1.33 | Infinity | 11.00 | 11.67 | Infinity | 3.08 | 4.92 | 1.44 | 1.08 | Infinity | 2.84 | Infinity |
|  | SPE (%) | 100.00 | 100.00 | 88.00 | 100.00 | 82.61 | 100.00 | 100.00 | 100.00 | 62.50 | 100.00 | 96.00 | 94.00 | 100.00 | 93.75 | 87.50 | 81.25 | 75.00 | 100.00 | 67.57 | 100.00 |
|  | SEN (%) | 16.00 | 60.00 | 40.00 | 31.11 | 47.83 | 42.50 | 76.56 | 40.63 | 50.00 | 24.00 | 44.00 | 70.00 | 19.23 | 19.23 | 61.54 | 26.92 | 26.92 | 7.59 | 92.00 | 40.00 |
|  | Cut-off | NA | NA | NA | NA | NA | NA | NA |  | NA | NA | NA | NA | NA | NA | NA | NA | NA | NA | NA | NA |
|  | Assay indicators | Methylation(P15) | Methylation (P16) | Mut（249Ser p53） | Methylation （p16INK4a） | Methylation （p16INK4A） | Methylation （RASSF1A） | Methylation (p16) | Methylation (DAPK) | Methylation (GSTP1) | Methylation(P15) | Methylation(P16) | Methylation (RASSF1A) | Methylation(P16) | Methylation (GSTP1) | Methylation（APC） | Methylation (RASSF1A) | Methylation (CDH1) | Mut (249Ser p53) | Methylation(p16INK4A) | Methylation（RASSF1A） |
|  | Detection methods | MSP |  | PCR | RTQ-MSP | MSP | MSP | MSP |  | MSP | MSP |  |  | MSP |  |  |  |  | RFLP | MSP | MSP |
|  | Sample source | Plasma and Serum |  | Plasma | Plasma | Serum | Plasma | Serum |  | Serum | Serum |  |  | Plasma |  |  |  |  | Plasma | Serum | Serum |
|  | Timing sample | Pre surgery |  | NA | Pre surgery | NA | NA | Next day of hospital |  | NA | Before diagnosis |  |  | NA |  |  |  |  | NA | NA | Prospective |
|  | HCC/BD/ HC | 25/35/20 |  | 45/30/20 | 45/30/20 | 46/23/- | 40/-/10 | 64/15/20 |  | 32/8/- | 50/-/50 |  |  | 26/16/- |  |  |  |  | 79/-/73 | 25/37/- | 35/-/10 |
|  | Control type | LC/HC |  | LC/HC | LC/HC | LC | HC | BD/HC |  | LC/HC | HC |  |  | LC |  |  |  |  | HC | LC/CAH | HC |
|  | Study type | Prospective |  | NA | Prospective | NA | NA | NA |  | NA | Prospective |  |  | NA |  |  |  |  | Case-control | NA | Retrospective |
|  | Country/ Region | Hong Kong |  | China | Hong Kong | Korea | Hong Kong | Nanjing |  | China | Taiwan |  |  | China |  |  |  |  | Nigeria | Egypt | China |
|  | Year | 2000 |  | 2003 | 2003 | 2004 | 2005 | 2005 |  | 2006 | 2007 |  |  | 2008 |  |  |  |  | 2008 | 2010 | 2010 |
|  | First author | Wong et al. |  | Huang et al. | Wong et al. | Chu et al. | Yeo et al. | Lin et al. |  | Wang et al. | Zhang et al. |  |  | Chang et al. | |  |  |  | Igetei et al. | Ahmed et al. | Hu et al. |

| Support table 1. Characteristics of the included studies. | TN | 31 | 35 | 31 | 41 | 24 | 22 | 119 | 75 | 37 | 43 | 23 | 31 | 33 | 33 | 68 | 19 | 71 | 43 | 368 | 112 |
| --- | --- | --- | --- | --- | --- | --- | --- | --- | --- | --- | --- | --- | --- | --- | --- | --- | --- | --- | --- | --- | --- |
|  | FN | 27 | 26 | 43 | 23 | 3 | 5 | 83 | 56 | 23 | 13 | 50 | 31 | 10 | 2 | 47 | 20 | 71 | 21 | 68 | 23 |
|  | FP | 6 | 2 | 6 | 9 | 3 | 5 | 14 | 13 | 6 | 0 | 14 | 6 | 1 | 1 | 13 | 27 | 6 | 14 | 26 | 8 |
|  | TP | 45 | 46 | 29 | 20 | 28 | 26 | 77 | 104 | 43 | 53 | 50 | 69 | 29 | 37 | 89 | 116 | 52 | 102 | 122 | 97 |
|  | NLR | 0.45 | 0.38 | 0.71 | 0.65 | 0.11 | 0.20 | 0.58 | 0.41 | 0.40 | 0.20 | 0.80 | 0.37 | 0.26 | 0.05 | 0.41 | 0.36 | 0.63 | 0.23 | 0.38 | 0.21 |
|  | PLR | 3.85 | 11.82 | 2.48 | 2.58 | 8.13 | 4.53 | 4.57 | 4.40 | 4.67 | #DIV/0! | 1.32 | 4.26 | 25.28 | 32.26 | 4.08 | 1.45 | 5.43 | 3.38 | 9.73 | 12.13 |
|  | SPE (%) | 83.78 | 94.59 | 83.78 | 82.00 | 88.89 | 81.48 | 89.47 | 85.23 | 86.05 | 100.00 | 62.16 | 83.78 | 97.06 | 97.06 | 83.95 | 41.30 | 92.21 | 75.44 | 93.40 | 93.33 |
|  | SEN (%) | 62.50 | 63.89 | 40.28 | 46.51 | 90.32 | 83.87 | 48.13 | 65.00 | 65.15 | 80.30 | 50.00 | 69.00 | 74.36 | 94.87 | 65.44 | 85.29 | 42.28 | 82.93 | 64.21 | 80.83 |
|  | Cut-off | NA | NA | NA | NA | NA |  |  | 400ng/mL | 5% (LOD) |  | NA | NA | MI > 0.88 |  | NA | >= 20 ng/ml | NA | 20 ng / ml | NA | 20 ng / ml |
|  | Assay indicators | Methylation (GSTP1) | Methylation（RASSF1A） | Methylation（SFRP1） | Methylation（TFPI2） | Methylation (DBX2) | Methylation (THY1) | methylation （TGR5） | TGR5 and AFP | methylation （INK4A） | INK4A and AFP | methylation（MT1M） | methylation（MT1G） | methylation（HOXA9） | HOXA9 or AFP | methylation（IGFBP7） | IGFBP7and AFP | methylation（CDO1） | CDO1 and AFP | methylation（RASSF1） | RASSF1 and AFP |
|  | Detection methods |  |  |  | MSP | Pyrosequencing |  | MSP |  | pyrosequencing |  | MSP |  | Q-MSP |  | MSP |  | MSP |  | MSP |  |
|  | Sample source |  |  |  | Serum | Serum |  | Serum |  | Serum |  | Serum |  | Plasma |  | Serum |  | Serum |  | Serum |  |
|  | Timing sample |  |  |  | NA | Pre treatment |  | NA |  | NA |  | Before therapy |  | During surgery |  | NA |  | NA |  | Before treatment |  |
|  | HCC/BD/ HC |  |  |  | 43/24/26 | 31/-/27 |  | 160/88/45 |  | 66/43/- |  | 121/37/- |  | 39/-/34 |  | 136/46/35 |  | 123/57/20 |  | 190/234/160 |  |
|  | Control type |  |  |  | CHB/HC | HC |  | CHB/HC |  | CLD/- |  | CHB/HC |  | HC |  | CHB/HC |  | LC/CHB/HC |  | LC/CHB/HC |  |
|  | Study type |  |  |  | Case-Control | Retrospective |  | NA |  | Retrospective |  | Retrospective |  | NA |  | Case-Control |  | Case-Control |  | NA |  |
|  | Country/ Region |  |  |  | China | China |  | China |  | America |  | China |  | Taiwan |  | China |  | China |  | China |  |
|  | **Year** |  |  |  | 2013 | 2013 |  | 2014 |  | 2014 |  | 2014 |  | 2014 |  | 2014 |  | 2014 |  | 2015 |  |
|  | **First author** |  |  |  | Sun et al. | Zhang et al. |  | Han et al. |  | Huang et al. |  | Ji et al. |  | Kuo et al. |  | Li et al. |  | Yang et al. |  | Dong et al. |  |

| Support table 1. Characteristics of the included studies. | | TN | | 8 | | 8 | 7 | | 6 | | 48 | | 57 | | 39 | | 228 | | 239 | | 241 | | 243 | | | 241 | | 59 | | | 70 | | 146 | | 76 | | 203 | | |  |  |  |
| --- | --- | --- | --- | --- | --- | --- | --- | --- | --- | --- | --- | --- | --- | --- | --- | --- | --- | --- | --- | --- | --- | --- | --- | --- | --- | --- | --- | --- | --- | --- | --- | --- | --- | --- | --- | --- | --- | --- | --- | --- | --- | --- |
|  |  | FN | | 13 | | 15 | 18 | | 19 | | 10 | | 34 | | 16 | | 47 | | 62 | | 69 | | 81 | | | 78 | | 27 | | | 37 | | 45 | | 22 | | 185 | | |  |  |  |
|  |  | FP | | 2 | | 2 | 3 | | 4 | | 52 | | 9 | | 27 | | 17 | | 6 | | 4 | | 2 | | | 4 | | 41 | | | 10 | | 27 | | 70 | | 53 | | |  |  |  |
|  |  | TP | | 18 | | 16 | 13 | | 12 | | 20 | | 77 | | 95 | | 51 | | 36 | | 29 | | 17 | | | 20 | | 53 | | | 43 | | 75 | | 98 | | 50 | | |  |  |  |
|  |  | NLR | | 0.52 | | 0.60 | 0.83 | | 1.02 | | 0.69 | | 0.35 | | 0.24 | | 0.52 | | 0.65 | | 0.72 | | 0.83 | | | 0.81 | | 0.57 | | | 0.53 | | 0.44 | | 0.35 | | 0.99 | | |  |  |  |
|  |  | PLR | | 2.90 | | 2.58 | 1.40 | | 0.97 | | 1.28 | | 5.09 | | 2.09 | | 7.50 | | 15.00 | | 18.13 | | 21.25 | | | 12.50 | | 1.62 | | | 4.30 | | 4.00 | | 1.70 | | 1.03 | | |  |  |  |
|  |  | SPE (%) | | 80.00 | | 80.00 | 70.00 | | 60.00 | | 48.00 | | 86.36 | | 59.09 | | 93.06 | | 97.55 | | 98.37 | | 99.18 | | | 98.37 | | 59.00 | | | 87.50 | | 84.39 | | 52.05 | | 79.30 | | |  |  |  |
|  |  | SEN (%) | | 58.06 | | 51.61 | 41.94 | | 38.71 | | 66.67 | | 69.37 | | 85.59 | | 52.04 | | 36.73 | | 29.59 | | 17.35 | | | 20.41 | | 66.25 | | | 53.75 | | 62.50 | | 81.67 | | 21.28 | | |  |  |  |
|  |  | Cut-off | | NA | | NA | NA | | NA | | NA | | NA | | 20 ng/ml | | PMR≥4% | |  | |  | |  | | |  | |  | | | 200ng/ml | | NA | | 20 ng/ml | |  | | |  |  |  |
|  |  | Assay indicators | | methylation（ELF） | | methylation（RASSF1A） | methylation（P16） | | methylation（GSTP1） | | methylation（SPINT2/HAI-2） | | Methylation（SOX11） | | SOX11 and AFP | | Methylation (RASSF1A)) | | Methylation (APC) | | Methylation (BVES) | | Methylation (GSTP1) | | | Methylation (HOXA9) | | Methylation (UBE2Q1) | | | UBE2Q1 and AFP | | Methylation (HCCS1) | | Methylation (HCCS1) and AFP | | Methylation (CDKN2A) | | |  |  |  |
|  |  | Detection methods | | MSP | |  |  | |  | | MSP | | MSP | |  | | MSP | |  | |  | |  | | |  | | MSP | | |  | | MSP | |  | | Pyrosequencing | | |  |  |  |
|  |  | Sample source | | Plasma | |  |  | |  | | Serum | | Serum | |  | | Serum | |  | |  | |  | | |  | | Serum | | |  | | Serum | |  | | Plasma | | |  |  |  |
|  |  | Timing sample | | NA | |  |  | |  | | NA | |  | |  | | NA | |  | |  | |  | | |  | | Prior to treatment | | |  | | NA | |  | | Before diagnosis | | |  |  |  |
|  |  | HCC/BD/ HC | | 31/10/- | |  |  | |  | | 30/50/50 | | 111/66/- | |  | | 98/165/80 | |  | |  | |  | | |  | | 80/80/20 | | |  | | 120/146/27 | |  | | 237/257/- | | |  |  |  |
|  |  | Control type | | HCC/LC | |  |  | |  | | LC/HC | | CHB/HC | |  | | LC/CHB/HC | |  | |  | |  | | |  | | LC/CHB/HC | | |  | | CHB/HC | |  | | BD | | |  |  |  |
|  |  | Study type | | NA | |  |  | |  | | Case-Control | | Retrospective | |  | | Case-Control | |  | |  | |  | | |  | | NA | | |  | | Case-Control | |  | | Case-Control | | |  |  |  |
|  |  | Country/ Region | | China | |  |  | |  | | Egypt | | China | |  | | China | |  | |  | |  | | |  | | China | | |  | | China | |  | | Taiwan | | |  |  |  |
|  |  | Year | | 2015 | |  |  | |  | | 2015 | | 2016 | |  | | 2017 | |  | |  | |  | | |  | | 2017 | | |  | | 2017 | |  | | 2017 | | |  |  |  |
|  |  | First author | | Huang et al. | |  |  | |  | | Ramadan et al. | | Teng et al. | |  | | Dong et al. | |  | |  | |  | | |  | | Hu et al. | | |  | | Tian et al. | |  | | Wu et al. | | |  |  |  |
| Support table 1. Characteristics of the included studies. | | TN | | 106 | | 63 | | | 114 | | 51 | | 147 | 201 | 20 | | 18 | | 30 | | 48 | | 24 | | 186 | 157 | | 79 | | 78 | 72 | | 60 | 33 | 110 | | 27 | | 47 | 20 | | 66 |
|  |  | FN | | 58 | | 50 | | | 3 | | 7 | | 85 | 112 | 27 | | 18 | | 5 | | 40 | | 38 | | 60 | 62 | | 27 | | 25 | 35 | | 25 | 12 | 66 | | 22 | | 22 | 13 | | 14 |
|  |  | FP | | 151 | | 17 | | | 21 | | 5 | | 10 | 12 | 4 | | 6 | | 0 | | 3 | | 27 | | 14 | 43 | | 21 | | 22 | 8 | | 26 | 21 | 12 | | 20 | | 28 | 1 | | 37 |
|  |  | TP | | 179 | | 105 | | | 48 | | 40 | | 34 | 37 | 29 | | 38 | | 16 | | 7 | | 9 | | 40 | 38 | | 73 | | 75 | 101 | | 80 | 93 | 52 | | 96 | | 96 | 23 | | 46 |
|  |  | NLR | | 0.59 | | 0.41 | | | 0.07 | | 0.16 | | 0.76 | 0.80 | 0.58 | | 0.43 | | 0.24 | | 0.90 | | 1.72 | | 0.65 | 0.79 | | 0.34 | | 0.32 | 0.29 | | 0.34 | 0.19 | 0.62 | | 0.32 | | 0.30 | 0.38 | | 0.36 |
|  |  | PLR | | 1.29 | | 3.19 | | | 6.05 | | 9.53 | | 4.49 | 4.41 | 3.11 | | 2.71 | | Infinity | | 2.53 | | 0.36 | | 5.71 | 1.77 | | 3.48 | | 3.41 | 7.43 | | 2.52 | 2.28 | 4.48 | | 1.91 | | 2.18 | 13.42 | | 2.13 |
|  |  | SPE (%) | | 41.25 | | 78.75 | | | 84.44 | | 91.07 | | 93.63 | 94.37 | 83.33 | | 75.00 | | 100.00 | | 94.12 | | 47.06 | | 93.00 | 78.50 | | 79.00 | | 78.00 | 90.00 | | 69.77 | 61.11 | 90.16 | | 57.45 | | 62.67 | 95.24 | | 64.08 |
|  |  | SEN (%) | | 75.53 | | 67.74 | | | 94.12 | | 85.11 | | 28.57 | 24.83 | 51.79 | | 67.86 | | 76.19 | | 14.89 | | 19.15 | | 40.00 | 38.00 | | 73.00 | | 75.00 | 74.26 | | 76.19 | 88.57 | 44.07 | | 81.36 | | 81.36 | 63.89 | | 76.67 |
|  |  | Cut-off | |  | | NA | | | ≥2 positive triplicates | | ≥2 positive triplicates | | NA | NA | NA | |  | | NA | | NA | | NA | |  |  | |  | |  | 20 ng/mL | |  | 400ng/ml |  | | 20ng/ml | | 20ng/ml | NA | | NA |
|  |  | Assay indicators | | Methylation (TBX2) | | Methylation (IGFBP7) | | | Methylation(mSEPT9) | | Methylation(mSEPT9) | | Methylation (SOCS3) | Mut-(TP53 R249S) | Methylation (FHIT) | | Methylation (P16) | | Methylation (EMX1) | | MUT-(TERT) | | Mut-(p53 R249S) | | Methylation (RASSF1A) | Methylation (SOCS1) | | RASSF1A and AFP | | SOCS1 and AFP | SEPT9 and AFP | | Methylation (CCND1) | CCND1 and AFP | Methylation (CCND2) | | CCND2 and AFP | | （Methylation）CCND2 | MUT-(TERT C228T) | | Methylation (mSEPT9) |
|  |  | Detection methods | |  | | MSP | | | real-time PCR | | real-time PCR | | MSP | DdPCR | MSP | |  | | MSP | | DdPCR | |  | | MSP |  | |  | |  |  | | MSP |  | MSP | |  | |  | WTB-PCR | | NGS |
|  |  | Sample source | |  | | Serum | | | plasma | | plasma | | plasma | plasma | serum | |  | | Plasma | | Serum | |  | | serum |  | |  | |  |  | | Plasma |  | Plasma | |  | |  | Serum | | Plasma |
|  |  | Timing sample | |  | | Thefirst day of diagnosis | | | Before diagnosis | | before diagnosis | | Before surgery | NA | NA | |  | | Before treatment | |  | |  | | NA |  | |  | |  |  | | Before treatment |  | NA | |  | |  | At the time of diagnosis | | Before treatment |
|  |  | HCC/BD/ HC | |  | | 155/60/20 | | | 51/135/- | | 47/56/- | | 119/157/50 | 149/164/49 | 56/-/24 | |  | | 21/30/- | | 47/-/51 | |  | | 100/100/100 |  | |  | |  |  | | 105/54/32 |  | 118/122/- | |  | |  | 36/21/- | | 60/103/- |
|  |  | Control type | |  | | CHB/HC | | | LC | | LC | | LC/BD/HC | CLD/HC | NC | |  | | LC | | HC | |  | | LC/HC |  | |  | |  |  | | CHB/HC |  | LC/CHB/HC | |  | |  | NH | | LC |
|  |  | Study type | |  | | NA | | | Observational | | Case-control | | Case-Control | Case-control | Case-control | |  | | Case-control | | Retrospective | |  | | Case-control |  | |  | |  |  | | Prospective |  | NA | |  | |  | Retrospective | | Case-control |
|  |  | Country/ Region | |  | | China | | | French | | Germany | | China | Cameroon or CAR | China | |  | | American | | Tunis | |  | | Egypt |  | |  | |  |  | | China |  | China | |  | |  | Japan | | American |
|  |  | Year | |  | | 2018 | | | 2018 | |  | | 2018 | 2018 | 2019 | |  | | 2019 | | 2019 | |  | | 2019 |  | |  | |  |  | | 2020 |  | 2020 | |  | |  | 2020 | | 2021 |
|  |  | First author | |  | | Li et al. | | | Oussalah(a) et al. | | Oussalah(b) et al. | | Wei et al. | Marchio et al. | Bai et al. | |  | | Kisiel et al. | | Marchio et al. | |  | | Pasha et al. |  | |  | |  |  | | Liu et al. |  | Qian et al. | |  | |  | Akuta et al. | | Lewin et al. |

| Support table 1. Characteristics of the included studies. | TN | 31 | 28 | 72 | 59 | 17 | 45 | 44 | 24 | 39 | 40 | 55 | 29 | 16 | 40 | 43 | 29 | 55 | 58 | 175 | 120 | 119 | 72 |
| --- | --- | --- | --- | --- | --- | --- | --- | --- | --- | --- | --- | --- | --- | --- | --- | --- | --- | --- | --- | --- | --- | --- | --- |
|  | FN | 38 | 16 | 4 | 14 | 7 | 22 | 41 | 38 | 47 | 18 | 22 | 7 | 1 | 10 | 17 | 6 | 16 | 9 | 67 | 30 | 45 | 50 |
|  | FP | 9 | 2 | 41 | 4 | 8 | 5 | 12 | 32 | 17 | 16 | 25 | 8 | 4 | 0 | 2 | 11 | 4 | 4 | 27 | 81 | 84 | 8 |
|  | TP | 41 | 36 | 59 | 49 | 18 | 38 | 67 | 70 | 61 | 90 | 58 | 65 | 71 | 30 | 22 | 39 | 24 | 15 | 121 | 157 | 144 | 86 |
|  | NLR | 0.62 | 0.33 | 0.10 | 0.24 | 0.41 | 0.41 | 0.48 | 0.82 | 0.62 | 0.23 | 0.40 | 0.12 | 0.02 | 0.25 | 0.46 | 0.18 | 0.43 | 0.40 | 0.41 | 0.27 | 0.41 | 0.41 |
|  | PLR | 2.31 | 10.38 | 2.58 | 12.25 | 2.25 | 6.33 | 2.90 | 1.13 | 1.86 | 2.92 | 2.32 | 4.18 | 4.93 | infinitely | 12.69 | 3.15 | 8.85 | 9.69 | 4.82 | 2.08 | 1.84 | 6.32 |
|  | SPE(%) | 77.50 | 93.33 | 63.72 | 93.65 | 68.00 | 90.00 | 78.57 | 42.86 | 69.64 | 71.43 | 68.75 | 78.38 | 80.00 | 100.00 | 95.56 | 72.50 | 93.22 | 93.55 | 86.63 | 59.70 | 58.62 | 90.00 |
|  | SEN (%) | 51.90 | 69.23 | 93.65 | 77.78 | 72.00 | 63.33 | 62.04 | 64.81 | 56.48 | 83.33 | 72.50 | 90.28 | 98.61 | 75.00 | 56.41 | 86.67 | 60.00 | 62.50 | 64.36 | 83.96 | 76.19 | 63.24 |
|  | Cut-off | 36.6 ng/mL | 73.0 ng/ml | NA | 1.50*105 copies/LAFP>20 | 0.56ng/ul | 1.87 × 104 copies/ uL | 0.2 pg/mL | 0.2 pg/mL | 0.2 pg/mL | 0.2 pg/mL | 98.2ng/ml | 18.2 ng/mL | NA | 304nmol -1 | 509 ng/mL | 8 copies/µl | 370 copies/mL | 30ng/ml | >0.4 | >0.1 | >0.2 | 4.6 copies |
|  | Assay indicators | ctDNA | Methylation (GSTP1) | Methylation (RASSF1A) | RASSF1A and AFP | ctDNA | hTERT | Methylation (BASP1) | Methylation (CCND2) | Methylation (CFTR) | Methylation (RASSF1A) | ctDNA | ctDNA | ctDNA and AFP | Methylation（RASSF1A） | ctDNA | Methylation （RASSF1A） | ctDNA | ctDNA | Methylation (RASSF1A) | Methylation (CDH1) | Methylation (RUNX3) | Methylation (SEPT9) |
|  | Detection methods | Ultraviolet transilluminator | Rt-PCR | Rt-PCR |  | QPCR | Rt-PCR | QMSP |  |  |  | QPCR | QPCR |  | RT-PCR. | NA | Rt- PCR | DdPCR | Qubit dsDNA | QMSP |  |  | DdPCR |
|  | Sample source | Plasma | serum | serum |  | serum | Plasma | Serum |  |  |  | serum | plasma |  | serum | serum | serum | plasma | plasma | serum |  |  | serum |
|  | Timing sample | Pre surgery | NA | At diagnosis |  | Before treatment | Pre treatment | NA |  |  |  | NA | before treatment |  | before treatment | NA | NA | NA | before therapy or surgery | NA |  |  | Before treatment |
|  | HCC/BD/ HC | 79/20/20 | 52/30/- | 63/63/50 |  | 25/25/- | 60/21/29 | 108/56/- |  |  |  | 80/80/- | 72/37/- |  | 40/40/- | 39/-/45 | 45/40/- | 40/29/30 | 24/62/- | 193/203/53 |  |  | 136/45/- |
|  | Control type | LC/HC | HCV-related chronic liver disease/HC | HBV infection/HC |  | CAH | HBV infection/ HC | HCV infection |  |  |  | CAH/HC | BD/HC |  | CAH/HC | HC | HCVinfection /HC | NH | LC | LC/HC |  |  | CLD/HC |
|  | Study type | NA | NA | NA |  | Prospective | NA | Prospective |  |  |  | NA | Case-Control |  | Retrospective | Case-Control | Case-Control | NA | NA | case–control |  |  | NA |
|  | Country/ Region | China | Japan | Hong Kong |  | Egypt | China | Japan |  |  |  | China | China |  | Egypt | China | Egypt | Hong Kong | China | Egypt |  |  | Japan |
|  | Year | 2006 | 2006 | 2008 |  | 2010 | 2011 | 2011 |  |  |  | 2012 | 2012 |  | 2012 | 2013 | 2017 | 2018 | 2018 | 2020 |  |  | 2020 |
|  | First author | Ren et al. | Lizuka et al. | Chan et al. |  | El-Shazly et al. | Yang et al. | Lizuka et al. |  |  |  | Chen et al. | Huang et al. |  | Mohamed et al. | Chen et al. | Mansour et al. | Gai et al. | Yan et al. | El-Bendary et al. |  |  | Kotoh et al. |

| TN | 314 | 246 | 165 |  | 146 | 139 | APC: Adenomatous polyposis coli;BASP1:brain abundant, membrane attached signal protein 1;BVES: blood vessel/epicardial substance; CAH：chronic acute hepatitis; CCND2: Cyclin D2; CDH1: Cadherin 1; CDO1, Cysteine dioxygenase 1;; CH, chronic hepatitis; CHB, Chronic hepatitis B; CLD, chronic liver diseases;CDKN2A: Cyclin dependent kinase inhibitor 2A; CFTR: Cystic fibrosis transmembrane conductance regulator; DBX2: Developing brain homeobox 2; DAPK：Death-associated protein kinase DdPCR: Droplet digital PCR; ELF: Spectrin beta, nonerythrocytic 1; EMX1, empty spiracles homeobox 1;FHIT: fragile histidine triad; FN: False negative; FP: False positive; GSTP1: Glutathione Stransferase pi 1; HOXA9: Homeobox A9; HCCS1 Hepatocellular carcinoma suppressor 1; HCC/BD/HC, number of hepatocellular carcinoma/benign live diseases/ healthy controls; IGFBP7, insulin-like growth factor-binding protein 7; M: Methylation; MSP: M specific PCR; MT1G: Metallothionein 1G; MT1M: Metallothionein 1M; Mut: Mutation; MSRE-QPCR: methylation-sensitive restriction enzymes-based quantitative PCR NA: Not available; NGS: Next-generation sequencies; NC: non-cancer control group p15: Cyclin dependent kinase inhibitor 2B; p16: Cyclin dependent kinase inhibitor 2A; p53: Tumor protein p53; RASSF1A: Ras association domain family member 1; RFLP: Restriction fragment length polymorphism; RT-PCR: Reverse transcription PCR; RUNX3: RUNX family transcription factor 3; SE: Sensitivity; SEPT9: Septin 9; SOCS: Suppressor of cytokine signaling ;SFRP1:  secreted frizzled-related protein 1;SP: Specificity; SPINT2/HAI-2: Serine peptidase inhibitor, Kunitz type 2; SRD5A2: Steroid 5 alpha-reductase 2; TBX2: T-box transcription factor 2; TERT: Telomerase reverse transcriptase; TGR5: G-protein-coupled bile acid receptor Gpbar1; TN, true negative; TP, true positive;TFPI2:tissue factor pathway inhibitor 2; THY1：Thy-1 cell surface antigen; UBE2Q1：ubiquitin-conjugating enzyme E2Q (putative)1；VIM: Vimentin；WTB-PCR：wild-type blocking PCR；ZNF154：zinc finger protein 154. |
| --- | --- | --- | --- | --- | --- | --- | --- |
|  |  |  |  | 143 |  |  |  |
| FN | 15 | 18 | 9 | 21 | 17 | 73 |  |
| FP | 28 | 23 | 9 | 17 | 14 | 1 |  |
| TP | 49 | 86 | 95 | 77 | 81 | 56 |  |
| NLR | 0.26 | 0.19 | 0.09 | 0.24 | 0.19 | 0.57 |  |
| PLR | 9.35 | 9.67 | 17.66 | 7.39 | 9.45 | 60.78 |  |
| SPE (%) | 91.81 | 91.45 | 94.83 | 89.38 | 91.25 | 99.29 |  |
| SEN (%) | 76.56 | 82.69 | 91.35 | 78.57 | 82.65 | 43.41 |  |
| Cu-toff | 41.1 | 41 | 20 ng/mL | 15.70% |  | 786432 |  |
| Assay indicators | Methylation (SePT9) | Methylation (SEPT9) | SEPT9 and AFP | ctDNA methylation ratio | ctDNA and AFP | Methylation (SGTP1) |  |
| Detection methods | QPCR | QMSP |  | DdPCR | QPCR | QMSP |  |
| Sample source | plasma | Plasma |  | Plasma |  | Plasma |  |
| Timing sample | Before therapy | NA |  | NA |  | NA |  |
| HCC/BD/ HC | 64/44/298 | 104/95/174 |  | 98/80/80 |  | 129/90/50 |  |
| Control type | LC/HC | BD/HC |  | CHB/HC |  | LC/CHB/HC |  |
| Study type | Case-control | Case-control |  | NA |  | NA |  |
| Country/ Region | China | China |  | China |  | China |  |
| Year | 2020 | 2020 |  | 2021 |  | 2021 |  |
| First author | He et al. | Li et al. |  | Wang et al. |  | Xie et al. |  |

A B


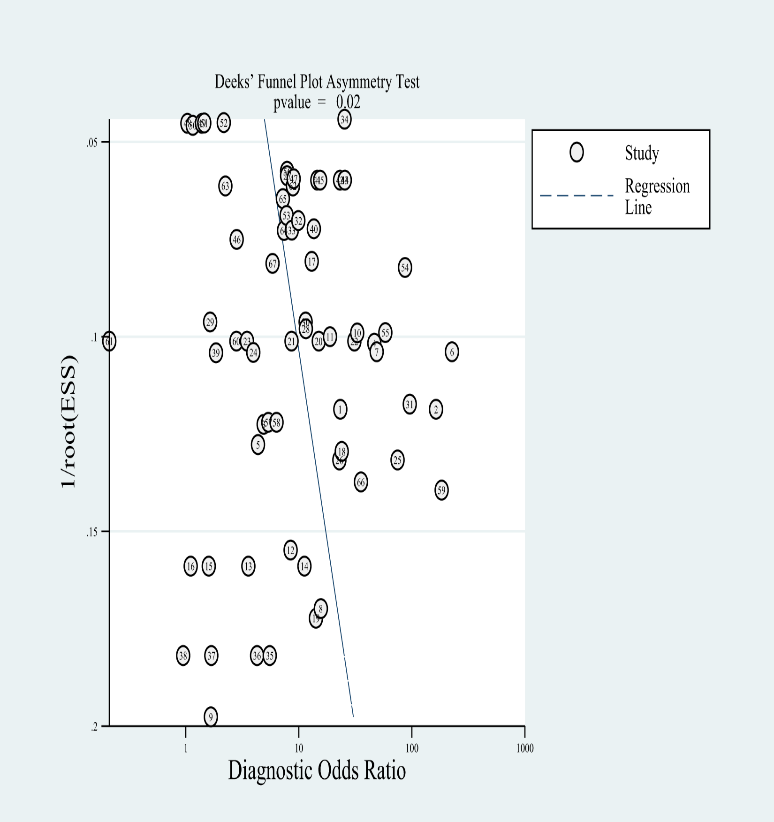

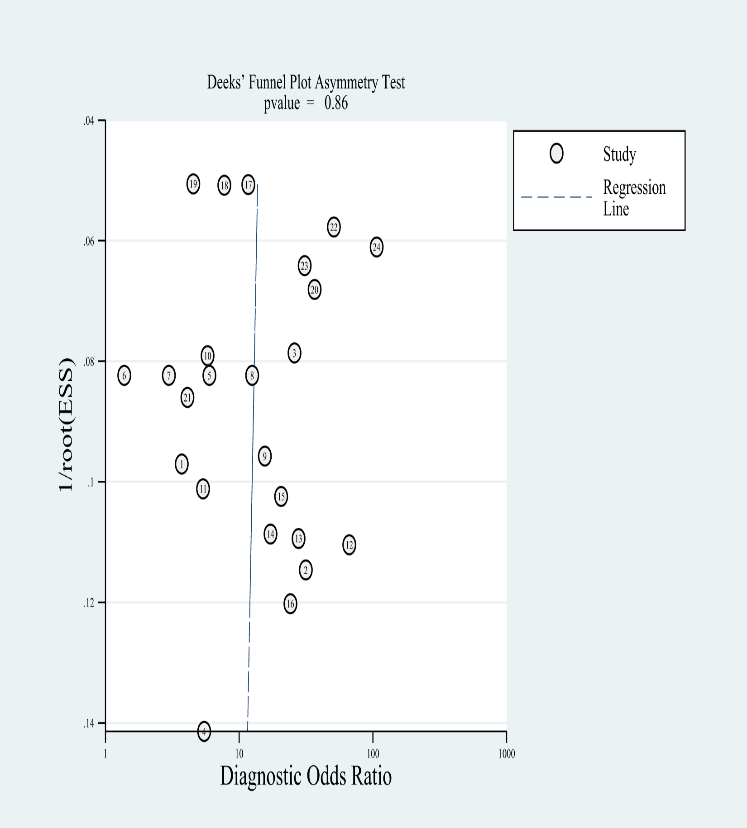

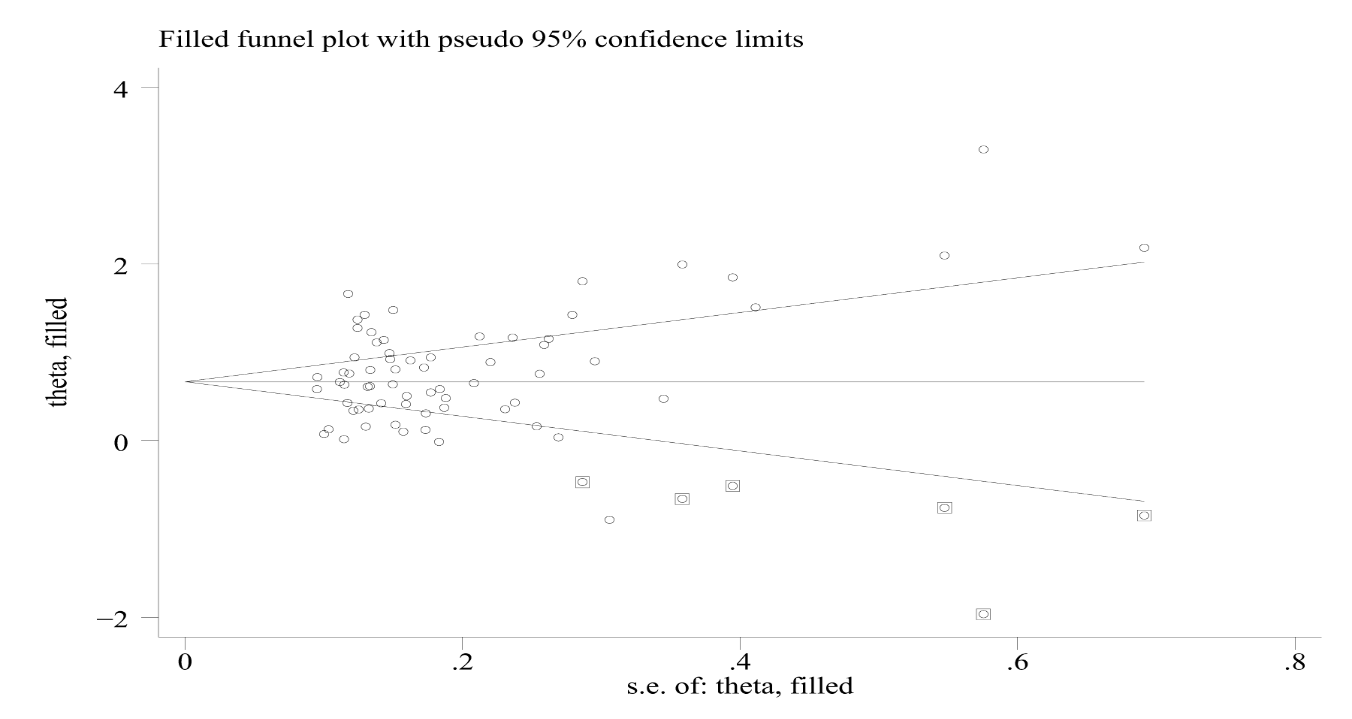


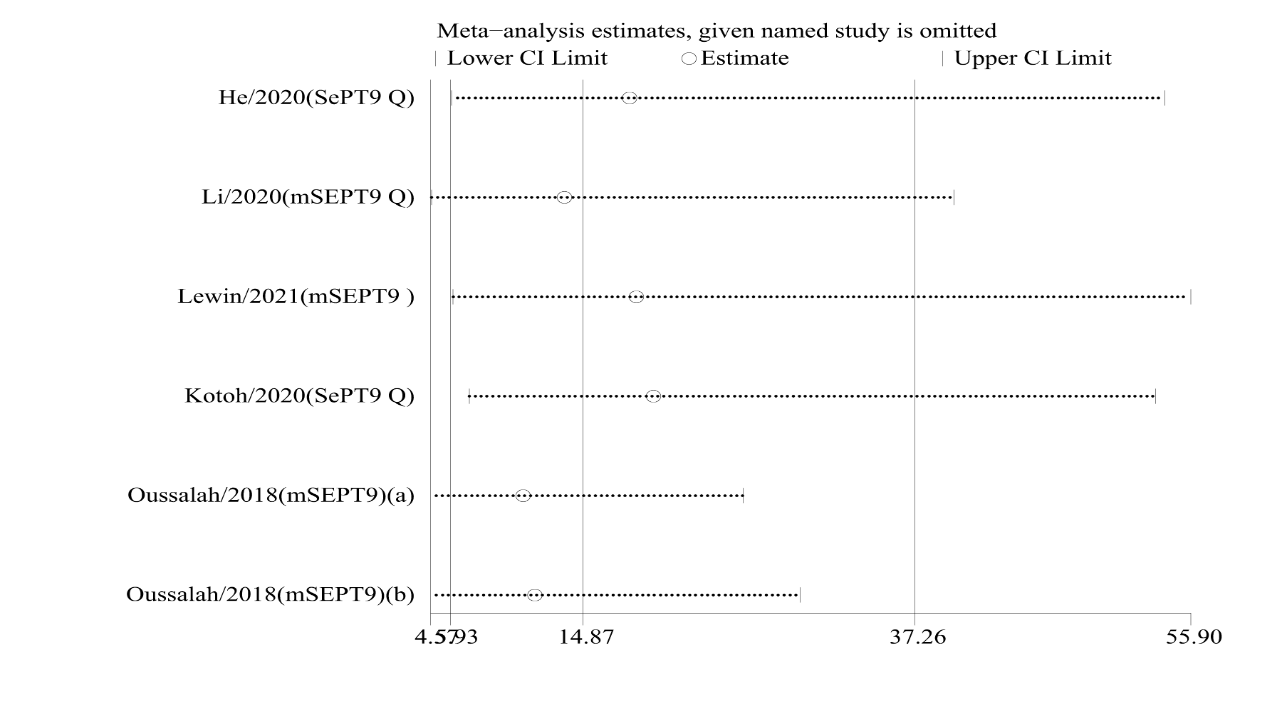

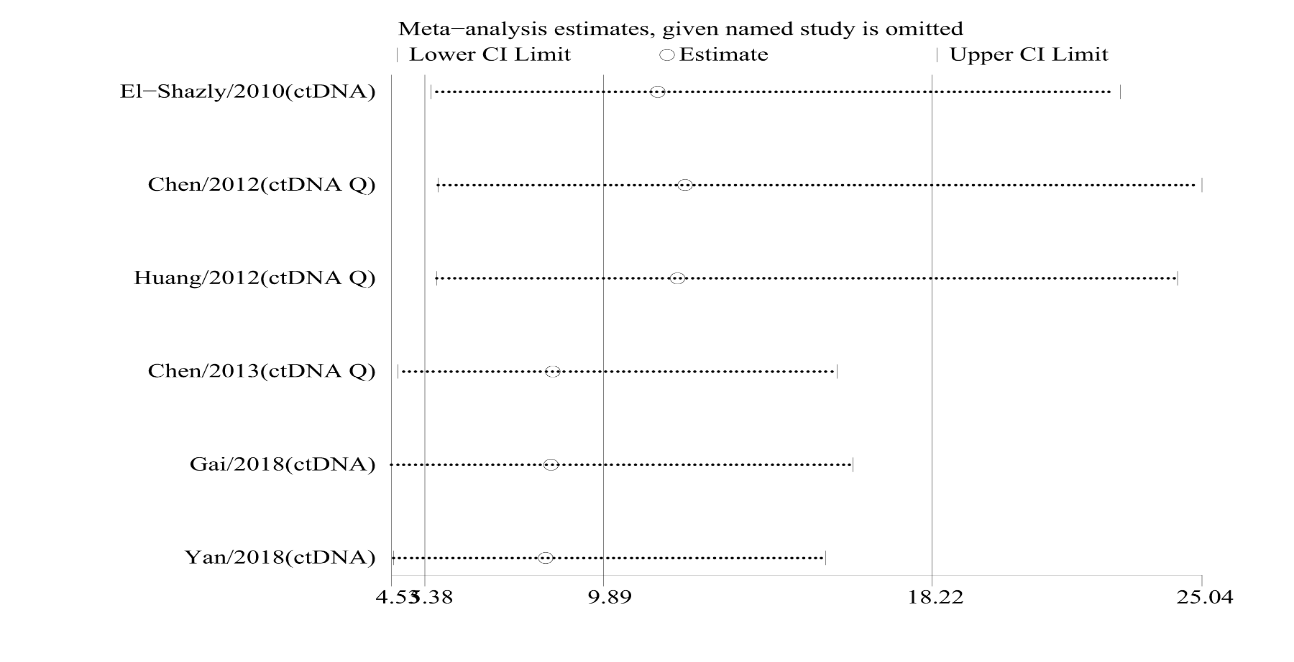


Figure 8. Publication bias analysis. (A) Deek’s funnel plot asymmetry test for qualitative studies. (B) Deeks funnel’ plot asymmetry test for quantitative studies. (C) Trim and fill analysis for qualitative studies. (D) sensitivity analysis of SETP9 methylation subgroup. (E) sensitivity analysis of ctDNA concentration subgroup

<https://gco.iarc.fr/today/fact-sheets-cancers>.

11 Leon SA, Shapiro B, Sklaroff DM, Yaros MJ. Free DNA in the serum of cancer patients and the effect of therapy. Cancer Res. ;37(3):646-50. PMID: 837366(1977).

12 Stroun M, Lyautey J, Lederrey C, Olson-Sand A, Anker P. About the possible origin and mechanism of circulating DNA apoptosis and active DNA release. Clin Chim Acta. 313(1-2):139-42. doi: 10.1016/s0009-8981(01)00665-9(2001) .

13 Huang, X. H. *et al.* Codon 249 mutation in exon 7 of p53 gene in plasma DNA: maybe a new early diagnostic marker of hepatocellular carcinoma in Qidong risk area, China. *World journal of gastroenterology* **9**, 692-695, doi:10.3748/wjg.v9.i4.692 (2003).
