# Supplementary figures and images for: Diagnostic performance of circulating tumor DNA as a minimally invasive biomarker for hepatocellular carcinoma: a systematic review and meta-analysis
Source: PeerJ. 2022 Nov 3;10:e14303. doi: 10.7717/peerj.14303 (PMC9637356; doi:10.7717/peerj.14303)

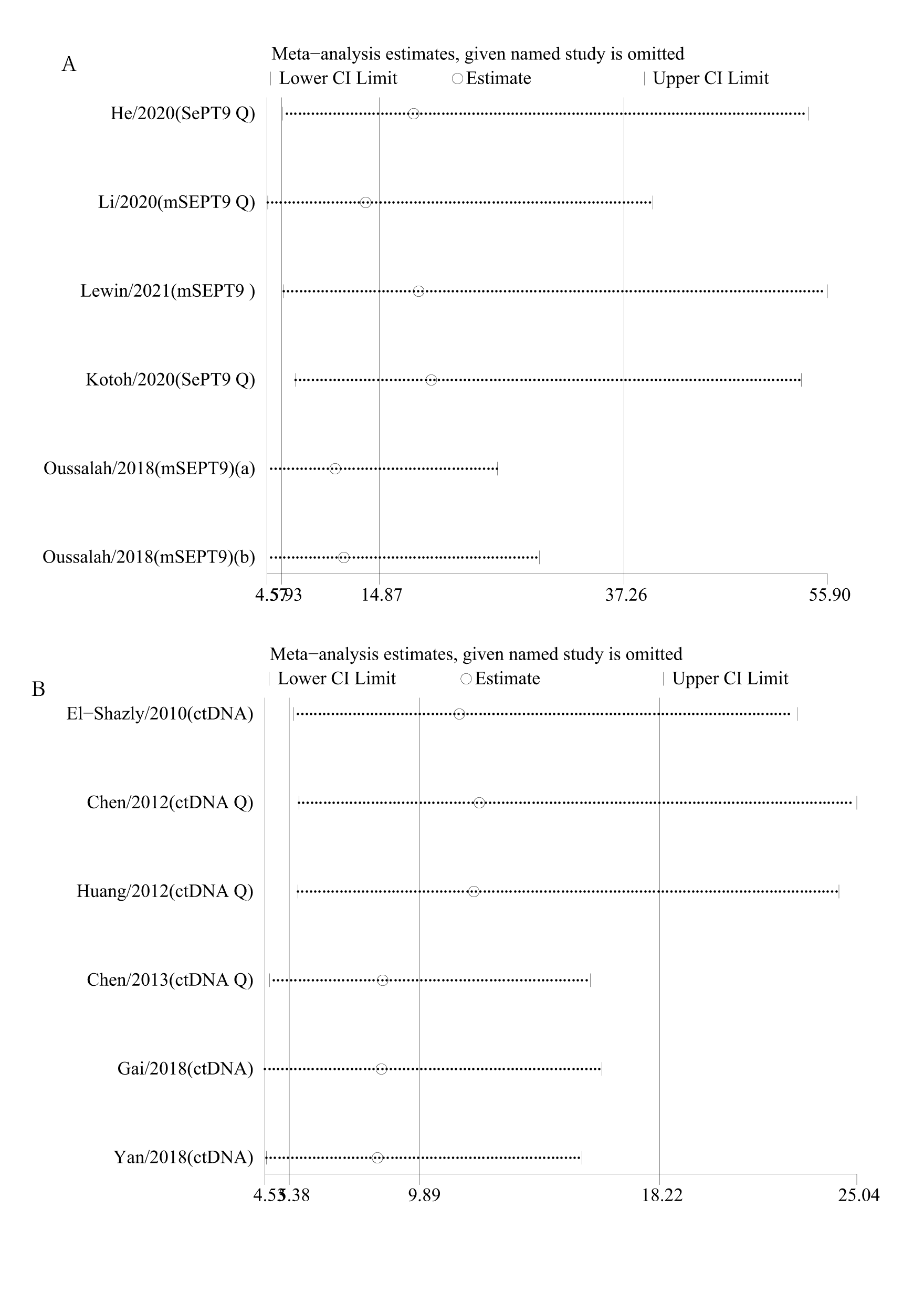

Supplement: Supplemental Information 7 [file peerj-10-14303-s007.png]
