# Supplementary material for: Diagnostic performance of circulating tumor DNA as a minimally invasive biomarker for hepatocellular carcinoma: a systematic review and meta-analysis
Source: PeerJ. 2022 Nov 3;10:e14303. doi: 10.7717/peerj.14303 (PMC9637356; doi:10.7717/peerj.14303)
Supplement: Supplemental Information 9 [file peerj-10-14303-s009.docx]

The rationale for conducting the systematic review / meta-analysis: (alpha-fetoprotein) AFP could not meet the need to diagnose hepatocellular carcinoma (HCC) in the early stage and we need non-invasive biomarkers to diagnose hepatocellular carcinoma independently or serve as a complementary of AFP in an affordable way. Circulating tumor DNA (ctDNA) potentially provide the chance to achieve this goal and a large number of studies have conducted to explore the possibility. However, we still lack large cohort study to support diagnostic performance of ctDNA. This meta-analysis supported the diagnostic value of ctDNA to some extent and proposed methylation SEPTIN9 (mSETP9) in ctDNA and ctDNA concentration may potentially diagnose HCC.

The contribution that it makes to knowledge in light of previously published related reports, including other meta-analyses and systematic reviews: compared with previously published related reports, we enrolled the studies in latest two years and enrolled studies that previous meta-analysis did not mention, which will allow a more comprehensive synthesis of the corresponding data. Besides, we analysis the diagnostic value of methylation SEPTIN9 (mSETP9) in ctDNA and ctDNA concentration, and performed sensitivity analysis to support the robustness of the results. In addition, we explore the latent source of heterogeneity in qualitative, quantitative or ctDNA concentration which potentially provides guidelines for the study of ctDNA with standardized processes
